# Supplementary material for: Functional Characterisation of the Rare SCN5A p.E1225K Variant, Segregating in a Brugada Syndrome Familial Case, in Human Cardiomyocytes from Pluripotent Stem Cells
Source: Int J Mol Sci. 2023 May 31;24(11):9548. doi: 10.3390/ijms24119548 (PMC10253753; doi:10.3390/ijms24119548)
Supplement: Supplementary file 1 [file ijms-24-09548-s001.zip › ijms-2382156-supplementary.pdf]

Supplementary Figures

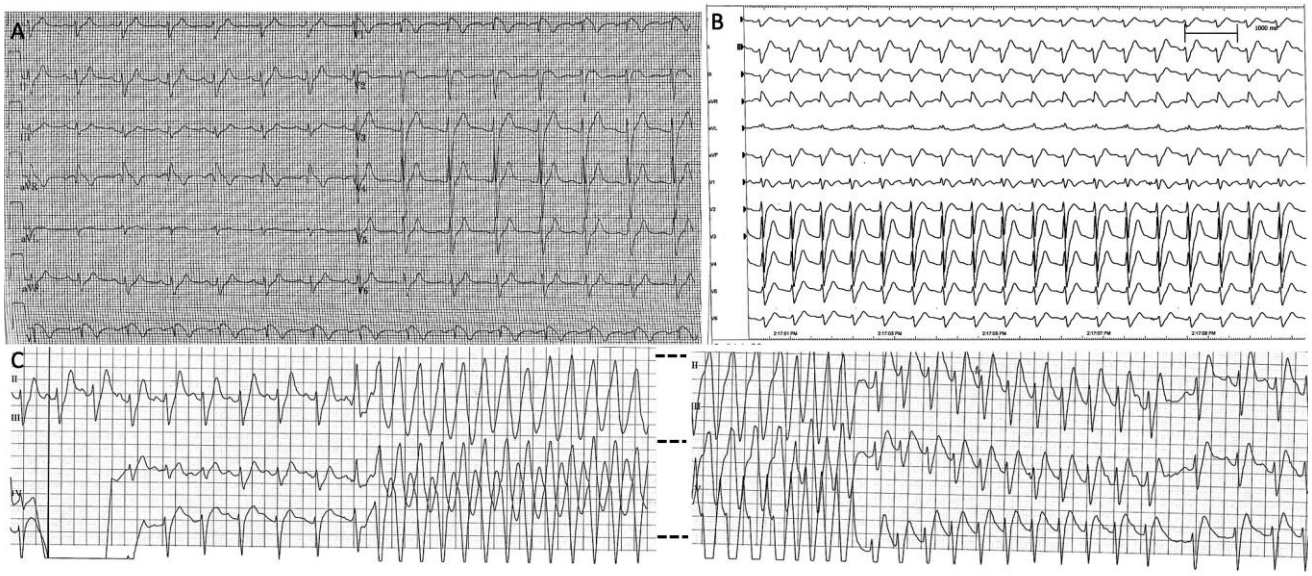

Supplementary Figure S1. Electrocardiographic patterns of the proband (II.2). (**A**) Baseline ECG, showing spontaneous type 1 Brugada pattern. (**B**) ECG pattern 10 minutes after flecainide challenge, showing Brugada pattern enhancement. (**C**) Polymorphic ventricular tachycardia (VT), due to flecainide toxicity.

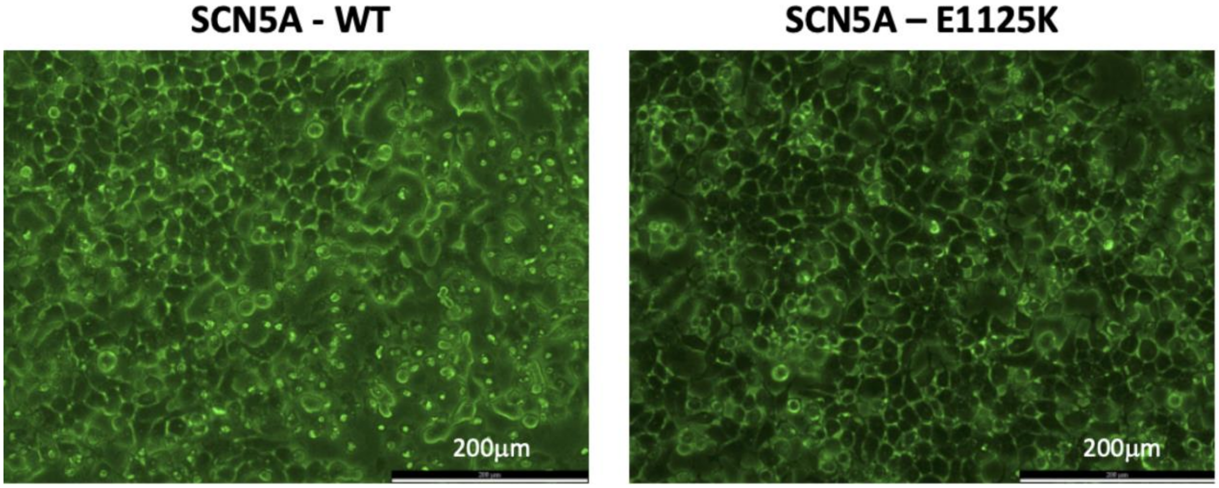

Supplementary Figure S2. Lentiviral particles production. Representative images of HEK293T cells transfected with the lentiviral constructs encoding *SCN5A* gene, either wt (left) or carrying the p.E1125K variant (right) for lentiviral particles production. Scale bar: 200 µM.
